# Consumers’ Motivational Involvement in eWOM for Information Adoption: The Mediating Role of Organizational Motives

**DOI:** 10.3389/fpsyg.2019.03055

**Published:** 2020-01-21

**Authors:** Safdar Hussain, Xi Song, Ben Niu

**Affiliations:** ^1^College of Management, Shenzhen University, Shenzhen, China; ^2^Pir Mehr Ali Shah, Arid Agriculture University Rawalpindi, Rawalpindi, Pakistan; ^3^Great Bay Area International Institute for Innovation, Shenzhen, China

**Keywords:** online opinions, consumer behavior, information quality, information usefulness, perceived risk, social tie

## Abstract

E-commerce offers an opportunity on web renounced in internet marketing, and the consumers’ communication behavior has changed, which has taken the place of word of mouth (WOM). This study investigated consumers’ motivational involvement in electronic word of mouth for online information adoption mediated by writers, motivations. Using a sample of 390 active Chinese internet users, it revealed that social tie and perceived risk are essential factors that influence consumers’ behavior, occur unpleasant consequences, and the possibility of uncertainties during the decision making process. Online retailers should emphasize perceived risk mitigation enable to provide a quick response on the websites. Practitioners need to understand consumer behavior in the online shopping system for the expansion of the online marketplace to product varieties, online advertising, retail strategies, and market segmentation. Organizations should train their service provides to timely response, concentrate on monitoring the aspects of consumers’ reviews, on creating choices among groups and individuals, which can improve the organization’s business performance.

## Introduction

With the emergence of internet technology from past decades, our lifestyles have altered considerably. The number of internet users is increasing rapidly, among 77.16 billion World’s population, 45.36 billion internet users were estimated in June 2019. Asian internet users contribute higher as 23.0 billion, which is 50.7% rest of the World’s population, while China determined 8.29 billion internet users that provide 37.7% among Asian internet users. E-commerce offers an opportunity in web renounced in internet marketing, and global e-retail sales are projected to reach 27 trillion U.S. dollars by 2020 in terms of online shopping (Cheung and Lee, 2012; Internet and Statistics, 2018). It is essential for practitioners and companies to understand consumers, behavior in the online shopping system for the expansion of the online marketplace to product varieties, online advertising, retail strategies, and market segmentation. Before the emergence of the internet technology, people share or obtain information from known individuals about product or service before decision making, which played an essential role in shaping consumers’ behavior, called word of mouth (WOM). Now, the consumers’ communication behavior has changed, which has taken the place of WOM in the form of discussion groups, blogs, or forums within popular networking sites, called electronic word of mouth (eWOM) (Hennig-Thurau et al., 2003; Voyer and Ranaweera, 2015).

The decision-making process is a multifaceted stage that influences customers directly or indirectly with different choices. Customers intend to seek information about products or services to maximize their satisfaction level, expectations, and experiences. Information adoption of recommended products or services is not only a one-time decision-making process but also for repeated use, first use, or pre-usage, which is a process that occurs consistently and over time (Blackwell et al., 2001; Comegys et al., 2006). In the context of online shopping, the vital community provides an opportunity to share or gain information to expose the quality and risk of products before decision-making. Consumers rely on online information provided by others, which may credible to adopt, may profoundly influence their behavior, subjective norms, beliefs, intention, and attitude. Involvement, information credibility, and information quality are important sources that appeal to consumers’ social ties as positive or negative eWOM (Sussman and Siegal, 2003; Plotkina and Munzel, 2016).

Word of mouth is a concept of informal and interpersonal communication with personal recommendations. WOM is a concept of informal and interpersonal communication with personal recommendations. WOM is an informal communication about products or services, acknowledged as a significant influence on people’s feelings and knowledge. WOM is in the oldest way to exchange or share information about products or services with known individuals, such as collogues, friends, and family members (Berger, 2014). Day (1971) and Brown and Reingen (1987) believed that WOM is a more effective way to attract than direct sales, advertising that lead to changing consumer’s attitude augmented by a higher degree of satisfaction, profitability, and loyalty. The power of WOM communications’ stimulus has recognized in prior studies that WOM found more persuasive, objectivity, expertness, and trustworthy compared personal selling, TV advertising, print ads, a radio which refers to traditional media (Herr et al., 1991).

With the emergence of internet technology, WOM communication extended to eWOM for further computer-generated settings. The comments provided by customers through the internet about product or services refer to eWOM, which impact on consumers’ buying behavior. Consumers post their comments, suggestions, reviews, and recommendations about product or services on the internet through weblogs, review sites, social networking sites, e-bulletin system, and discussion forums or platforms, etc. (Hennig-Thurau et al., 2004; Cheung and Lee, 2012). Cheung and Thadani (2012) and Yan et al. (2016) stated that the dimensions of eWOM communication are different from traditional WOM, such as unique scalability, diffusion speed, while conventional WOM refers to sharing information among known individuals within private conversations way. eWOM communication is more accessible, persistent, measurable compared to traditional WOM, and involves multi-way information exchanges at the same time. In other words, consumers can retrieve many online messages to reduce their risk and uncertainties and enhance trust during decision making.

Information Adoption Model (IAM) integrated that the adoption or rejection of the information depends on the individual’s intention, beliefs, and behavior. Venkatesh and Davis (2000) extended IAM that describes usage intentions and apparent usefulness in terms of that explains apparent usefulness and usage intentions in terms reasoning and influential social processes referred to the assessment of adoption’s concerns with similar manners. Consumers strongly believe in information usefulness that determines them in terms of message notices to provide social cognitions, judgments, and choices (Sussman and Siegal, 2003). Whereas, TRA and TAM provide useful evidence of behavioral intentions in terms of information adoption, but has a limited scope of influence process, as well as Erkan and Evans (2016) claimed that IAM deal with information quality and source credibility that influence on information adoption. It is a need to integrate IAM and TRA to investigate the characteristics of information use that influence on consumers’ behavioral intentions.

**FIGURE 1:**
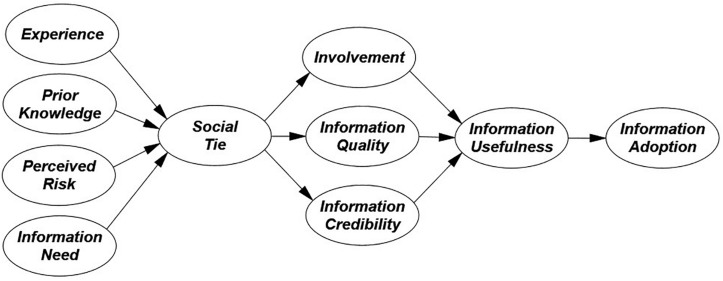
The conceptual model.

Most of the studies, such as Hennig-Thurau et al. (2003), Sweeney et al. (2012), and Hussain et al. (2018) focused on factors analysis, antecedents, motivations, platforms, recognitions that influence on consumers’ purchasing adoption or intention. However, there is still a gap available that needs to focus on the reader’s motivational involvement. Secondly, Hsu and Nien (2008) and Han and Kim (2017, pp 25–26) justified that Chinese online consumers are more ethnocentric than other Asian online users because of high uncertainty avoidance adoption to tend exhibit anxiety and risk reduction in online marketplaces. Furthermore, this study purposed social ties as an essential mediator that concentrate on consumer’s perception among eWOM seeking information shared on online platforms that leads to information adoption positively or negatively. The current study focused on reader’s motivations that involve in online communities, such as experience, prior knowledge, perceived risk, and information need, in response to eWOM information adoption, mediated by writer’s motivations, such as a social tie, involvement, information credibility, information quality, and information usefulness. So, this study purposed to determine the reader’s motivational factors that influence on Chinese consumers’ information adoption behavior in online communities (see Figure 1), as presented by intended by Sussman and Siegal (2003) and Erkan and Evans (2016), with the following objectives:

• To explore how social-ties influence the motivational participation of the readers.
• To examine the impact of a social tie on information credibility, involvement, and information quality influenced by information usefulness.
• To determine the impact of information usefulness in response to eWOM information adoption in online communities.
• To explore how experience, prior knowledge, and perceived risk positively affect social tie, while information need has a negative influence on social-tie.
• To examine a relationship between Information usefulness and online consumer’s information adoption.

### Literature Review and Hypotheses Development

In recent times, consumers’ psychology and their buying behavior at online platforms are becoming interesting to understand, and increasingly it has become complicated in the current global market. Therefore, in a broader view of the psychologically driven consumers, motivation and consumer decision-making process features are critical for consumers’ online information adoption in making their purchase decisions (Suki, 2016; Martínez-Ruiz et al., 2018). The existing literature on consumer psychology and organizational motives evidenced vast contributions during the last two decades, and earlier studies have provided a lot of information on consumers’ judgment of various brands in decision making for online shopping and information adoption. The existing empirical literature on consumers’ behavior in decision-making through online information adoption is comprehensive and demonstrates highly domain-specific consequences. The marketing 4.0 perspective has emerged by the start of 2017, and its primary goas are helping business organizations to reach and psychologically motivate their consumers’ comprehensively than in the previous years’ investigations to analyze the shifts in online and traditional consumer behavior (Kotler et al., 2016). It presents marketing 3.0 evaluation, which refers to technologies used as a way to know, interaction, and communication for establishing with prospective consumers. The use of technology helps consumers’ information adoption for purchasing online products, primarily through abundant online shopping possibilities, which are critical and revolutionizing the online information adoption environment, and consequently, it helps consumers to seek relevant information.

Over the past decade, the advancement of new technologies and the Internet has transformed both online marketing platforms and consumer psychology (Martínez-Ruiz et al., 2018). The existing scientific literature related to online information consumption environment highlights of numerous organizations adapting these mentioned innovative tools and strategies. Computer-generated reality (Charron, 2017), promotional activities and online services through web pages use (Blazquez-Resino et al., 2016; Méndez-Aparicio et al., 2017), mobile advertising, which provides consumers’ online information (Hongyan and Zhankui, 2017; Martínez-Ruiz et al., 2017), and the use of eWOM (Huete-Alcocer, 2017). However, there is a literature gap in identifying how online customers’ psychologically consume online information and perceive brands to make their decisions for purchase. However, this research on online information adoption and its impacts on customers’ psychology in decision-making is insightful and beneficial for future investigators in a broader perspective (Jost, 2017). It integrates numerous empirical findings into the purchase intention of consumers’ psychology through a comprehensive framework on online information adoption (Martínez-Ruiz et al., 2018). The proposed model of this study provides an integrative framework, which focuses on the relationship between predictor variables and consumers’ information adoption, and this model presents new constructs to broaden the understanding of online consumers’ psychology, which affects decision-making.

#### Social Tie

The set of social interactions occurring between two or more people presents the term of a social tie. Earlier studies identified that social relations are critical and play a significant role in the decision-making process of consumers (Arndt and Foundation, 1967; Steffes and Burgee, 2009). Previous literature documented different types of social ties, and researchers have investigated their effect on consumers’ decision-making. Granovetter (1973) described the concept of social tie strength, which shows the closeness of the relationships among individuals. The advantage of social ties ranges from strong to weak. The link of fragile social relations among individuals refers to acquaintances, and such people do not know each other intimately. Various marketing researchers have investigated the effects and importance of social ties concerning the decision-making process of consumers (Arndt and Foundation, 1967; Pasternak, 2017).

Strong or weak and social relations have different impacts on consumers’ purchasing decision-making processes, such as stronger social ties of friends exert a stronger influence over a receiver buying decisions than a weak relationship of acquaintances (Brown and Reingen, 1987). Information adoption by online customers affects consumers’ decision-making. The research study provides a piece of empirical evidence. It suggests that online customers’ motivational involvement in eWoM for information adoption by considering organizational motives is essential to process, and it affects consumers purchasing decision-making. The study shreds of evidence that online social relationships are vital and provide adequate measures for analyzing consumer behavior. Besides, literature evidence indicated that theories describing relations and social ties on eWOM communication and interaction support the model of this proposed study. Previous studies were instrumental in enhancing the understanding of different review aspects; however, these studies overlooked several exciting features of consumer behavior of online consumers’ information adoption. The majority of earlier studies, generally, ignored the social process, which could underlie online consumers’ adoption of electronic word-of-mouth (eWOM) information.

#### eWOM Organizational Motives

Electronic word of mouth is a form of influential communication that influences on individual customers and organizations’ perspectives. Argument quality, source credibility, eWOM valance, information usefulness, review quantity, and information adoption impacted as an eWOM response during decision making (Sussman and Siegal, 2003; Cheung et al., 2008). Consumers seek information from the informative and authentic source, and more online reviews as available on online communities and platforms (Cheong and Morrison, 2008). eWOM valence strongly impacts consumers’ behavior positively or negatively, because online reviews enhance the awareness, attitude, and consideration of product and services. Aggarwal et al. (2012) revealed that negative eWOM influence more significant on consumers’ attitudes than comparing to positive eWOM, especially when all the reviews are negative; however, more positive eWOM pay more attention for purchase intention.

Electronic word of mouth is a source of appealing for customers, and it increases the product and brand reputation, as well as it’s a route of social interaction while gathering information about product or services (Lu et al., 2013; Nieto et al., 2014). Social interaction, understanding, substitutes, promotions, offering coupons, and consumers’ involvement in products, such as restaurants, hotels, movies, CDs, and books can enhance retailers’ sales substantially (Bai et al., 2017). Nieto et al. (2014) and Bai et al. (2017) explored that rating and review numbers affect consumers’ purchase decisions, improve business performance, such as business owners’ market perceptions, profitability, and satisfaction.

External websites play more role for customers’ information seeking or searching process than internal sites as hosted by retailers because of proliferation, additionally, eWOM product and quality sentiments considered by customers, it is necessary for retailers to improve it to compete in the successive market (Liang et al., 2015). As business performance and knowledge transfer are essential in eWOM organizational behavior, business strategy is also a critical factor that plays a significant role in eWOM to dynamically communicate with customers by replying online reviews associated with management. Retailers’ business strategies can improve to demonstrate eWOM through opinion leaders by using their knowledge, reputation, and experiences, as providing helpful information, which can enhance the product’s sale with good ratings and attract the customers (see Table 1).

**TABLE 1:** eWOM organizational vs. consumers’ perspectives.

**Factors**	**Characteristics**	**References**
Argument quality	Appropriate, reliable, up-to-date, values, applicable, fulfill a need, relevant, complete, accurate, consistent	Bailey and Pearson, 1983; Wu and Shaffer, 1987; Sussman and Siegal, 2003
Source credibility	Knowledgeable, expertness, homophily, objectivity, trustworthiness,	Cheung et al., 2009; Xie et al., 2011; Hu, 2015; Hussain et al., 2017
Information usefulness	Valuable, helpful, informative	Racherla and Friske, 2012; Cho and Sagynov, 2015; Babiæ Rosario et al., 2016; Iyer et al., 2017
Response to eWOM	The intention, willingness, attitude, adoption, perceived credibility, perceived usefulness, actual use or purchase	Choi and Geistfeld, 2004; Clemes et al., 2014; Hennig-Thurau et al., 2015; Erkan and Evans, 2016

#### Hypotheses Formulation

***H1.*** Experience, and perceived risk have a positive influence on the social tie, whereas, information need, and prior knowledge have a negative impact on a social tie.
***H2.*** The social tie has a positive influence on involvement, and information quality, while information credibility negatively impacted by social tie.
***H3.*** Information quality and information credibility are strictly related to having a positive impact on information usefulness, whereas involvement has adverse effects on information usefulness.
***H4.*** There is a close relationship between Information usefulness and online consumers’ information adoption.

## Materials and Methods

### Participants

This research study organized a self-administered questionnaire and distributed it among recruited 545 Chinese active internet users; screened collected forms by testing the data received by using the SPSS-24 version. However, the active 390 respondents were selected as recommended by Krejcie and Morgan (1970), to measure the instrument for personal identification that influences on Chinese consumers’ information adoption behavior in online communities as presented by Sussman and Siegal (2003) and Erkan and Evans (2016). The respondents were requested to rate their level of willingness to adopt online information in online communities through a questionnaire (five-points Likert scale). For this study, about 53.8% participated were selected females (210 members), while 46.2% respondents were males (180 members), and their ages were between 21 and 35 years (62%). This study participants selected who used the internet frequently, read online reviews and comments, and shopped online. The qualification of the majority of participants was graduation (36.6%) with high frequency, the occupation of the high respondents was associated with companies, sales or services (47.5% in total). Concerning the income range, 57.4% had an income ranging from 3000 to 8000 RMB, more than 75% respondents’ search for products or services on the internet before decision making.

### Measurements

#### Experience, Prior Knowledge, Perceived Risk, and Information Need

The variables of reader’s motivational involvement, such as experience, prior knowledge, perceived risk, and information need were measured through paradigms as developed by Wu and Shaffer (1987), Hennig-Thurau et al. (2004), Huang et al. (2009), Jeong and Jang (2011), Martin et al. (2015), Hussain et al. (2017), and Yang (2017). Pre-usage (before experience), first use (first experience), and repeated use (experience-oriented) are stages of the adoption process for the experience of desired products or services. Pre-usage or before the experience, consumers obtain information from experienced reviewers or opinion leaders that make them more persuasive and credible (Bettman and Park, 1980; Huang et al., 2009). In online opinion leadership, people believe themselves as a leader and think people regard them as a good source of information; many people give high weight about their opinions on the internet (Sun et al., 2006).

Perceived risk is about consumers’ feeling about unpleasant consequences and the possibility of uncertainties, play a critical role during the decision making. Perceived risk varies the degree of reviews and extremity that has an antagonistic relationship with information related interaction due to negative reviews extremely or moderately toward retailers or brand websites. So, consumers seek data/information from a different source to reduce the risk when decision making (Kim et al., 2005). Because of significant alternatives, people try to manage their risk due to facing actual uncertainties. Information seekers consider them as not familiar with product, service, or topic, perceive risk and uncertainties, and find the information from others’ experiences, which might be useful for them to taking action. Hennig-Thurau et al. (2004) anticipated that information need is a notion of reader’s motivational aspect that consumer engages in eWOM, used as advice seeking or opinion seeking from opinion leaders or judgment given in online communities. Consumers search or request for the information on shopping websites to fulfill their desired information provided by others, considering information useful and adaptable lead to information adoption (Wolny and Mueller, 2013).

#### Social Tie and eWOM Involvement

The variable “social tie” was measured through paradigm as constructed by Brown and Reingen (1987) and Steffes and Burgee (2009), while the variable “involvement” was measured through paradigm as drawn up by Park and Lee (2008) and Voyer and Ranaweera (2015). Information obtained from reliable tie connection sources is more influential than information gained from weak tie connection sources. The advantage of social ties ranges from strong to weak. The relationship of a weak social tie among individuals refers to acquaintances, and such people do not know each other intimately. In the real situation, consumers seek the information and opinions from others to reduce their perceived risk, which they consider more relevant, trustworthiness, objectivity, ease of use that information provided by marketers (Laczniak et al., 2001).

There are many motivational involvements that consumers involve in eWOM, such as physical involvement, social involvement, self-worth reinforcement involvement, product involvement, economic involvement, other involvement, and brand involvement, etc. (Khammash and Griffiths, 2011; Hussain et al., 2018). Due to limited time availability, it is difficult for consumers to visit the markets to obtain information individually, so that, they prefer to achieve the desired information on virtual communities to save their time. Product information, the use of the product, product’s advantages, availability of new products in the market, latest product trends involve the consumers to engage in eWOM because of the new market phenomenon. Additionally, consumers involve in eWOM because of social relationships among others to compare the same experienced products or services, as well as to acquire some economic incentives, such as coupons, remunerations, points, discounts as monetary benefits (Hennig-Thurau et al., 2004; Hussain et al., 2018). People encounter a substantial degree of eWOM information, probably to choose the ideas as provided by virtual communities (Erkan and Evans, 2016). Consumers’ engagement in eWOM detailed info is more likely to have a significant impact on information adoption behavior. In virtual communities, consumers tend to seek the opinions posted by others before making the right decision (Cheung and Thadani, 2012).

#### Information Quality, Information Credibility, Information Usefulness, and eWOM Information Adoption

The variables “information quality,” and “information credibility” were measured through paradigms as developed by Wu and Shaffer (1987), Citrin (2002), Gupta and Harris (2010), Xie et al. (2011), whereas, “information usefulness,” and “eWOM information adoption” measured through paradigms as constructed by Sussman and Siegal (2003), Racherla and Friske (2012), Cho and Sagynov (2015), Erkan and Evans (2016), and Zhu et al. (2016) by using elaboration likelihood model (ELM) in response to consumers’ online involvement in eWOM for purchasing decision. Directly or indirectly on computer-mediated forums, argument quality, and source credibility get more attention to tend and determine the information, whether it is useful or not (Cheung et al., 2008).

Information quality refers to the central cue of information perceived by a message receiver, which influences the consumers’ attitude change, information adoption, and behavioral intentions. Bhattacherjee and Sanford (2006) defined information quality as “the persuasive strength of arguments embedded in an informational message.” Information source credibility belongs to peripheral cues of information recipients as a credible source with the degree to communicator’s assertion. As information source credibility, the degree of positive characteristics of information provider influence on recipients’ confidence, believe, trustworthiness, and competent, which lead to acceptance of a message (Cheong and Morrison, 2008). Likewise, if the information provided by a high source that may change the recipient’s views with the high significance of acceptance, the opinions, considering more useful and reliable, while low credibility makes less significance of message advocate.

Sussman and Siegal (2003) and Cheung and Lee (2012) predicted that within the online platform, information usefulness or perceived usefulness is also an individual perception by using new ideas and opinions which enhance the performance articulation about product or services. It is a user adoption’s predictor for system usage with strong associations. People think that if the comments, online reviews, and opinions posted by opinions leaders or experienced persons within online platforms are useful that can influence grater for information adoption.

### Data Analysis

The study was investigated as casual for interference minimization in the non-contrived environment from the customers of the People’s Republic of China, who used the internet to seek information about products or services for eWOM information adoption. Individuals were selected who used the internet as a unit of analysis to accumulate the data. Data was gathered cross-sectional for review by using questionnaire method (five-points Likert scale); non-probability purposive sampling technique was used as sample design. To check data reliability, Cronbach’s alpha (α) and average variance extracted (AVE) were used to measure internal consistency through SPSS, and the amount of variance by construct related to set of items, and measurement errors. Confirmatory factor analysis (CFA) was used for factor analysis to measure the constructs’ consistency, as recommended by Hoyle (2000). Structural equation modeling (SEM), as presented by Byrne (2016) represents in this study for casual path analysis, the regression weight, and model fit summary of the conceptual model.

## Results

This research study directed descriptive analysis to identify the demographics’ information adoption behavior of internet users considering organizational motives who read the reviews or comments before purchasing decisions. Tavakol and Dennick (2011) calculated that the scale reliability refers to a psychometric test related to each item, which measures through Cronbach’s alpha to estimate the internal consistency, the value ranges between 0.7 and 01 are acceptable. Table 2 shows that Cronbach’s alpha values of experience, prior knowledge, perceived risk, and information need are 0.920, 0.920, 0.895, and 0.931, while social tie, information quality, involvement, information credibility, information usefulness, and information adoption had values 0.844, 0.963, 0.940, 0.936, 0.845, 0.086, by using SPPS which are considered acceptable. For AVE as constructed by Hulland (1999), four factors of reader’s motivations with 16 items, and other six factors to adopt online information with thirty items were extracted to measure the amount of variance, resulted 0.75, 0.76, 0.69, 0.82, 0.56, 0.70, 0.79, 0.70, 0.64, and 0.68. The composite reliability (CR) was also measured, the values of each construct are over 0.70, which are acceptable because of the above the thresholds value (Brunner and Süβ, 2005). The values of composite reliability resulted experience, prior knowledge, perceived risk, and information need are 0.92, 0.93, 0.91, and 0.93, while social tie, information quality, involvement, information credibility, information usefulness, and information adoption had values 0.83, 0.94, 0.96, 0.94, 0.86, and 0.81, respectively (see Table 2).

**TABLE 2:** Confirmatory factor analysis, Cronbach’s alpha, average variance extracted, and composite reliability (*n* = 390).

**Constructs**	**Items**	**Factor loadings**	**α**	**AVE**	**CR**	**Constructs**	**Items**	**Factor loadings**	**α**	**AVE**	**CR**
Experience	EX1	0.972	0.920	0.75	0.92	Social tie	ST1	0.805	0.844	0.56	0.83
	EX2	0.830					ST2	0.811			
	EX3	0.917					ST3	0.698			
	EX4	0.718					ST4	0.654			
Prior knowledge	PK1	0.920	0.920	0.76	0.93	Information quality	IQ1	0.936	0.940	0.70	0.94
	PK2	0.936					IQ2	0.812			
	PK3	0.853					IQ3	0.969			
	PK4	0.764					IQ4	0.774			
Perceived risk	PR1	0.945	0.895	0.69	0.91		IQ5	0.808			
	PR2	0.698					IQ6	0.822			
	PR3	0.804					IQ7	0.698			
	PR4	0.759				Information Credibility	IC1	0.875	0.936	0.70	0.94
	PR6	0.850					IC2	0.841			
Information need	IN1	0.879	0.931	0.82	0.93		IC3	0.961			
	IN2	0.932					IC4	0.826			
	IN3	0.905					IC5	0.872			
Involvement	IT1	0.963	0.963	0.79	0.96		IC6	0.639			
	IT2	0.909					IC7	0.799			
	IT3	0.848				Information usefulness	IU1	0.808	0.845	0.64	0.84
	IT4	0.735					IU2	0.757			
	IT5	0.947					IU3	0.832			
	IT6	0.898				Information adoption	IA1	0.829	0.806	0.68	0.81
	IT7	0.914					IA2	0.822			

Discriminant validity is one of the theoretical approaches which provides the measurements of constructs that should not be highly associated with each other. Table 3 present the uniqueness of measures for each construct which explain the discriminant validity, argued by Fornell and Larcker (1981) and Stratman and Roth (2002). Convergent and discriminant validity was measured from the AVE and SCR values for each construct. The calculated values for AVEs are exceeded than 0.5, and also SCR values were greater than 0.7. The diagonal letters with boldfaces of each construct presented in Table 3 show the square root of AVEs, assessed from the percentages of shared common variance for each item, the values of inter-correlations were lower than AVE values, which supported the model (see Table 3).

**TABLE 3:** | The discriminant validity (*n* = 390).

**Construct**	**IN**	**PK**	**EX**	**PR**	**ST**	**IT**	**IQ**	**IC**	**IU**	**IA**
Information need	**0.91**									
Prior knowledge	−0.03	**0.87**								
Experience	0.02	0.02	**0.87**							
Perceived risk	−0.01	0.01	0.33	**0.83**						
Social tie	−0.02	0.02	0.44	0.34	**0.75**					
Involvement	−0.03	0.01	0.53	0.49	0.47	**0.89**				
Information quality	−0.07	0.03	0.10	0.06	0.11	0.11	**0.84**			
Information credibility	−0.32	0.00	0.01	0.04	0.01	0.06	0.17	**0.84**		
Information usefulness	−0.07	−0.01	0.10	−0.01	0.04	−0.03	0.25	0.28	**0.80**	
Information adoption	−0.02	−0.04	−0.04	0.02	0.00	0.01	0.08	0.19	0.11	**0.83**

*The diagonal letters with boldfaces of each construct show the square root of AVEs.*

**FIGURE 2:**
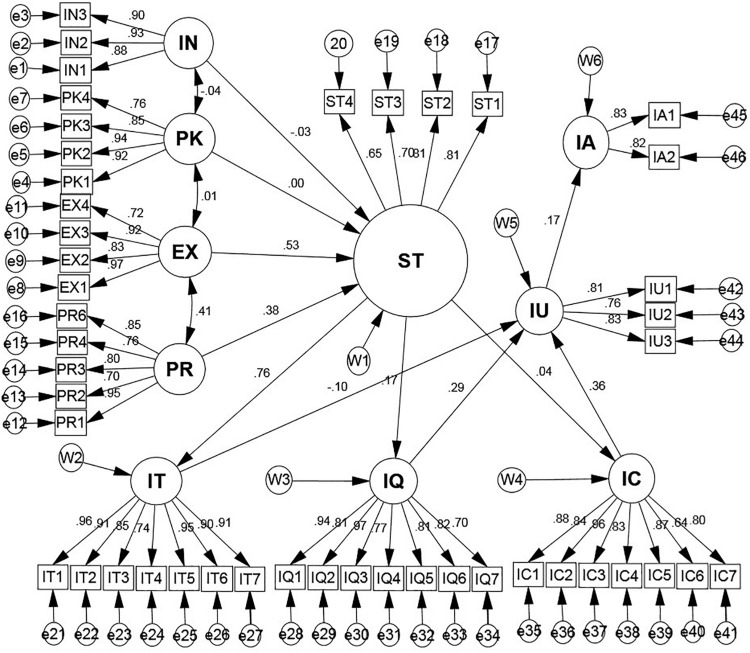
The structural model: IN, information need; PK, prior knowledge; EX, experience; PR, perceived risk; ST, social tie; IT, involvement; IQ, information quality; IC, information credibility; IU, information usefulness; IA, information adoption.

Figure 2 presents the SEM technique, which was used to combining CFA for casual path analysis, the regression weight, and model fit summary of the conceptual model. The structural model shows the relationship between the variables. The *p*-value indicated 0.000 because *p*-value presents the appropriate assumptions for model fitness in the population entirely, there is a likely relationship against the hypotheses with the level of significance (5%) (Jöreskog, 1969). The acceptable values of chi-square/df are ranging from 1 to 3, as recommended by Mciver and Carmines (1981). Table 4 presents the Chi-square/df value, which is 2.166, indicated goodness of the model. The values of *p* (*p*-value), RMSEA (root mean square of approximation), GFI (goodness of a fit index), AGFI (adjusted goodness of fit index), TLI (Tucker-Lewis coefficient), NFI (normed fit index), and CFI (comparative fit index) are 0.000, 0.055, 0.811, 0.890, 0.925, 0.876, and 0.929, which indicated the benchmark values and supported not absolute fit but moderate goodness of the model, because of slightly lower values compare to good model fit (Browne and Cudeck, 1993; Jöreskog and Sörbom, 1993; Hair et al., 2009) (see Figure 2 and Table 4).

**TABLE 4:** Model fit indices.

**Factors**	**Values**	**Factors**	**Values**
CMIN	2112.176	GFI	0.811
DF	975	AGFI	0.890
CMIN/DF	2.166	TLI	0.925
*p*-Value	0.000	NFI	0.876
RMSEA	0.055	CFI	0.929

Table 5 shows the regression weights estimation of each casual path, standard errors, critical ratios, *p*-values, and results were examined as per the conceptual framework. The findings of this survey suggested that social context favorable views such as friends, colleagues, family members, and relatives motivate Chinese consumers to buy products and services and it has an influence on consumers purchase intention consistent with socialists’ high motivation in complying with preference groups, which forms collectivistic key features (Triandis, 2000; Zhang and Khare, 2009). The reader’s motivational involvement in eWOM, such as experience, and perceived risk have a positive influence on social tie with the estimated values of 0.391, and 0.295, while with the estimated value (−0.020) of information need, and prior knowledge (0.003) have a negative impact on a social tie because of insignificance results. Social tie has more significant influence on information quality, and involvement with the estimated values of 0.197, 0.959, whereas, information credibility has insignificant influence on social tie (038), information quality, and information credibility positively supported information usefulness evaluated value ranges of 0.400, and 0.276, while involvement with the estimated value (−0.078) did not help by information usefulness, whereas, information usefulness (0.166) positively impacted by eWOM information adoption (see Table 5).

**TABLE 5:** The regression weights of casual paths (*n* = 390).

**Factors**	**Estimates**	***SE***	**Critical ratio**	***p*-Values**	**Results**
Social tie ← Information need	−0.020	0.024	−0.831	0.406	Not Supported
Social tie ← Prior knowledge	0.003	0.025	0.112	0.911	Not Supported
Social tie ← Experience	0.391	0.038	10.281	^∗∗∗^	Supported
Social tie ← Perceived Rrisk	0.295	0.038	7.840	^∗∗∗^	Supported
Involvement ← Social tTie	0.959	0.081	12.851	^∗∗∗^	Supported
Information quality ← Social tie	0.197	0.062	3.178	0.001	Supported
Information credibility ← Social tie	0.038	0.052	0.743	0.457	Not Supported
Information usefulness ← Information quality	0.276	0.050	5.476	^∗∗∗^	Supported
Information usefulness ← Involvement	−0.078	0.040	−1.935	0.053	Not Supported
Information usefulness ← Information credibility	0.400	0.064	6.221	^∗∗∗^	Supported
Information adoption ← Information usefulness	0.166	0.063	2.627	0.009	Supported

*^∗∗∗^*p*-Values < 0.05.*

## Discussion

This study sought out that the reader’s motivation hypotheses “experience,” and “perceived risk” have a positive influence, while “information need,” and “prior knowledge,” revealed a negative impact on “social tie.” It is difficult for product or services providers to fulfill the consumers’ desires and need because sharing information between inexperienced and experienced customers may cause the degree to perceived risk and uncertainty. Negative experiences influence more on consumers’ behavior, refer to dissatisfaction, disappointment, switch to other responses, because after unsatisfactory experiences react and discuss with others, which lead product or services failure. The eWOM communication process starts with the development of emotions and attitude toward products or services with the eWOM sources, such as feelings, knowledge, and experiences. eWOM source and pre-existing knowledge of receiver are the indicators with eWOM topics and platforms that provide valid eWOM prior knowledge, experience, and familiarity. Inexperienced customers consider other people as source expertise and follow their advice or opinions that impact on their purchase intention. Martin and Lueg (2013) examined that eWOM source with high expertise has more influence on brand attitudes and quality compared to low expertise, refers to trust or accept eWOM with the internet experience.

Social tie activation, information seeking, perceived influence, subgroups, and overlaps are the significant components that influence consumers’ behavior during information processing. Consumers feel that experiencing with product or service quality, atmosphere, and price fairness triggering them for positive strength tie. Positive experiences refer to consumers’ trust or accept eWOM with internet experience. In terms of prior knowledge, Chinese consumers take part in online communities to get objective, subjective knowledge, to share past experiences and prior visitation, which may reduce their physical, psychological, and performance risk, and influence on their purchasing decision. It has been witnessed from previous studies that experiences and prior knowledge are multidimensional constructs, which are essential factors that influence on consumers’ risk perception concerns, objective, subjective knowledge, and trust during information search (Bettman and Park, 1980; Sharifpour et al., 2014; Karimi et al., 2015). Reviews and comments reading on online platforms help them to reduce uncertainty, decrease unpleasant experiences concerns, and increase their confidence.

Uncertainties and taking risks are the factors that force consumers to take part in eWOM concerning personality traits, brand loyalty, and brand performance to alter their risk perceptions. It is essential for online retailers to understand consumers’ perceived uncertainties, which refer to a mode of purchase, the amount at stake, place of purchase, and individuals’ feelings. Perceived risk is an essential component of decision making so that online retailers need to identify customers’ risks related to transactions, products, purchasing modes, source, time, privacy, and social aspects. Information need has negative impact on a social tie and results match with the findings of Steffes and Burgee (2009) because consumers think when they try to apply new product consideration, negative eWOM messages perceptions occur in their minds by leading of weak tie connection sources. Because of little product and services experience on the internet, consider not more relevant, trustworthiness, objectivity, ease of use that the information need. It is essential for reviewers to build strong bonds with consumers in online forums to provide accurate information for reducing their uncertainties, risks (Kim et al., 2011).

The hypothesis “social tie” has a positive influence on “involvement,” and “information quality,” whereas, “information credibility” has a negative influence on social tie. Product information, the use of the product, product’s advantages, availability of new products in the market, latest product trends involve the consumers to engage in eWOM, and the results endorse as analyzed by Hussain et al. (2018). Consumers consider the online information shared is more precise, understandable with high quality. The source comprised valid arguments about online reviews perceived by recipients that influence their potential buying behavior. Consumers think that the reference provided by reviewers is credible, convincing, reliable, and accurate because information obtained from reliable tie connection sources is more influential. The strength of strong and weak ties are essential spectrums ranging from active primary to weak secondary, such as close friends, family members, colleagues, and contacted acquaintances. Mizerski (1982) provided evidence that tie activation, information seeking, perceived influence, subgroups, and overlaps are the significant components that influence consumers’ behavior during information processing. Negative eWOM has a more substantial impact on consumers’ behavior during the brand evaluation as compared to positive eWOM. Marketers believe that negative eWOM messages influence on receiver more than positive eWOM.

“Information quality” and “information credibility” have a significant favorable influence on “information usefulness,” while “involvement” has an adverse effect on information usefulness. Consumers believe that popular online reviews forms or websites provide more useful information, many reviews help them to evaluate the product’s performance. Registered reviewers on websites represent usefulness of experiences and knowledge about product or services. Trustworthiness, objectivity, and homophily are the elements which refer to consumers believe with the same situation, pronouns, honesty, length of contents, emotions, unusual events, age, gender, interest, and the same way to adopt the information. Information source credibility plays a substantial role during the information process, which saves the consumers’ time and avoids the confusions tending to judge the information for the acceptance or rejection of online source (Xie et al., 2011). Moreover, eWOM source credibility depends on the perception and judgment of the receiver’s evaluation and objectives of accuracy, subjective perception and information quality perceived by online reviews, comments, and messages. Trustworthiness, homophily, objectivity, expertise, and attractiveness are the major components of eWOM source credibility that narrates to the source’s reliability, believability, beneficial, honesty and make valid assertions for inferences and interest with a positive attitude (Hu, 2015; Hussain et al., 2017).

Negative or positive attitudes toward information and source comprise valid or invalid arguments about online reviews perceived by recipients that influence potential buying behavior. Negative online reviews affect consumers’ perceived reliability and familiarity because consumers consider that negative eWOM reviews are more useful as compared to positive eWOM reviews. Users make sure and evaluate the information quality entrenched in comments, measured by consistency, accuracy, relevance, trustworthiness, completeness, and timeliness dimensions of information providers. These dimensions denote the degree of resemblance, evaluation, resolve the uncertainty and ambiguity problems, output information precision, informative and sufficient information when intending to buy products and services online. The information shared by others provides them relevant, up to date, understandable, clear, applicable, reliable information that completes their sufficient need and included necessary values. Furthermore, information usefulness has a significant favorable influence on eWOM information adoption because consumers think online information is more useful, helpful, and informative to eWOM information adoption, make more accessible to decision making, and enhance their effectiveness. If the comments, online reviews, and opinions posted by opinions leaders or experienced person within online platforms are useful than can influence grater for information adoption.

## Conclusion

The primary focus of the study explored the relationship between predictor variables through a mediating effect on consumers’ information adoption in making their online purchase decisions. The prevailing literature related to online information consumption highlights organizations adapting such advanced tools and strategies. Computer-generated authenticity (Charron, 2017), promotions and online services by web pages usage (Blazquez-Resino et al., 2016; Méndez-Aparicio et al., 2017), advertising that offers online consumers’ latest information (Hongyan and Zhankui, 2017; Martínez-Ruiz et al., 2017), and the usage of eWOM (Huete-Alcocer, 2017). Though the literature gap exists, and it needs to identify how online customers’ psychologically seek online information and perceive various brands to make purchase decisions. E-commerce provides an opportunity for web renounced in internet marketing. The decision-making process is a multifaceted stage that influences customers directly or indirectly with different choices. Customers intend to seek information about products or services to maximize their satisfaction level, expectations, and experiences. Consumers rely on online information provided by others, which may credible to adopt, may highly influence their behavior, subjective norms, beliefs, intention, and attitude. It is difficult for product or services providers to fulfill the consumers’ desires and need because sharing information between inexperienced and experienced customers may cause the degree to perceived risk and uncertainty.

The findings illustrate the contributions to understand the Chinese consumers’ online information adoption behavior linked between consumers and e-commerce industries. It may help retailers and marketers to develop technological advancements, to make proper market strategies to attract current and new customers for valid and continuous offers and to strengthen the competitive advantages. It is essential for practitioners and companies to understand consumer behavior in the online shopping system for the expansion of the online marketplace to product varieties, online advertising, retail strategies, and market segmentation. Organizations should train their service to provide a timely response, concentrate on monitoring the aspects of consumers’ reviews, on creating choices among groups and individuals, which can improve the organization’s business performance. Companies need to build concern and trust among online critical reviews, identify eWOM disseminators, review systems, formulating strategies, and operational use of eWOM. The corporate response needs to careful consideration not only valence but also information and context that may perceive by customers.

This study indicates that social tie is the most substantial variable that influences on consumers’ behavior, the strength of strong and weak ties are essential spectrums ranging from active primary to weak secondary. It has been observed that negative online reviews affect consumers’ perceived reliability and familiarity because consumers consider that negative eWOM reviews are more useful as compared to positive eWOM reviews. Further, it revealed that perceived risk is an essential factor that consumers think whether shopping mode is safe or not, more concern about security risks, privacy, and payment method during personal information handling. Information seekers consider them as not familiar with product, service, or topic, perceive risk and uncertainties, and find the information from others’ experiences, which might be useful for them to taking action. Online retailers should emphasize perceived risk mitigations enable to provide a quick response on the websites.

This research work also has some limitations, like any other study. This study was someway limited to its experimental design. The predictor variables influenced consumers’ information adoption through the selected variables mediation, and these were the part of consumers’ overall experience. Therefore, various factors such as consumers’ prior experience, knowledge, perceived risk, information need, previous attitude, new products or services information, product preference, social tie, consumers’ involvement, credibility, usefulness, and quality of information affect purchasing decision-making. Thus, future studies might consider the impact of individual personal characteristics, for instance, services or product knowledge, product preferences, and consumers’ involvement. This specific study demonstrated that predictor variables such as consumer experience, perceived risk, information need, information, and social tie impacted information adoption by online shopping consumers. Future studies might examine other likely mediating variables such as consumers’ attitudes toward services or products, which might enhance the relationship between purchase intention and information sources quality and credibility. Besides, this study has not examined the impact of negative suggestions, endorsements, and information. Future researchers may investigate the influences contrary approvals and online information adoption. Previous literature evidenced that negative and positive information exerts a distinct impact on consumers’ behaviors (Charlett et al., 1995; Dellarocas, 2003). Scholars may examine the effects of various recommendations on consumers’ decision-making process through online information adoption. Future studies should focus on reviewing social risks, psychological risks, physical and performance risks (Jacoby, 2002; Hirunyawipada, 2006).

This study enlisted and examined some factors of the reader’s motivational involvement in analyzing consumers’ reactions toward eWOM. However, researchers might focus on other critical factors such as price ease of use, consciousness, and influence of different peer groups. The findings of this specific study are limited to this university of online Chinese consumers’ only, which presented their perception to compare reader’s motivational involvement in response to eWOM. Future studies might focus other factors and examine their effects on online buying consumers in different regions of the world with even larger sample size by testing other mediating variables such as consumers’ purchase behavior, prior attitude, online opinion seeking, brand trust and loyalty, and interpersonal influence, which might affect online consumers’ behavior. The previous literature has documented limited factors covering eWOM organizational and individuals’ conscious; however, scholars should examine other external factors such as subjective norms and generation. Previously, numerous research studies adapted quantitative methods and used instruments taken from various studies; however, the qualitative approach could also produce exciting results to understand the phenomenon of behavioral intention by adopting a panel study. Different studies such as Hennig-Thurau et al. (2004), Erkan and Evans (2016), and Hussain et al. (2017) focused on analysis and recognition aspects, antecedents and consequences, adoption and intention that influence on consumer behavior, however, visual eWOM, empirical credibility, text to speech, and continuing behavioral blocks need to be considered in the future that might influence the consumer.

## Data Availability Statement

The datasets generated for this study are available on request to the corresponding author.

## Ethics Statement

The studies involving human participants were reviewed and approved by the Board of Studies and Research of College of Management, Shenzhen University, Shenzhen, China. The patients/participants provided their written informed consent to participate in this study.

## Author Contributions

SH developed the conceptual notions and drafted and revised the manuscript. XS contributed in literature, methods, analysis, and revision. BN reviewed the revised manuscript critically, provided substantial contributions, and approved the final version to be submitted.

## Conflict of Interest

The authors declare that the research was conducted in the absence of any commercial or financial relationships that could be construed as a potential conflict of interest.
